# Hospital-Acquired Infection Underlies Poor Functional Outcome in Patients with Prolonged Length of Stay

**DOI:** 10.1155/2013/312348

**Published:** 2013-08-14

**Authors:** Alexander J. George, Amelia K. Boehme, James E. Siegler, Dominique Monlezun, Bethena D. Fowler, Amir Shaban, Karen C. Albright, T. Mark Beasley, Sheryl Martin-Schild

**Affiliations:** 1 Stroke Program at Tulane University Hospital, Department of Neurology, 1440 Canal Street, TB-52, Suite 1000, New Orleans, LA 70112-2715, USA; 2 Department of Epidemiology, School of Public Health, University of Alabama at Birmingham, 1665 University Blvd., Birmingham, AL 35294-0022, USA; 3 Department of Neurology, School of Medicine, University of Alabama at Birmingham, 1670 University Blvd., Birmingham, AL 35233-0022, USA; 4 Health Services and Outcomes Research Center for Outcome and Effectiveness Research and Education (COERE), 1530 3rd Avenue South, Medical Towers, Birmingham, AL 35294-4410, USA; 5 Center of Excellence in Comparative Effectiveness Research for Eliminating Disparities (CERED) Minority Health & Health Disparities Research Center (MHRC), Medical Towers Building, 1717 11th Avenue South, Suite 516A, Birmingham, AL 35294-4410, USA; 6 Department of Biostatistics, School of Public Health, University of Alabama at Birmingham, 1665 University Blvd., Birmingham, AL 35294-0022, USA

## Abstract

**Introduction:**

Prolonged length of stay (pLOS) following ischemic stroke inflates cost, increases risk for hospital-acquired complications, and has been associated with worse prognosis.

**Methods:**

Acute ischemic stroke patients admitted between July 2008 and December 2010 were retrospectively analyzed for pLOS, defined as a patient stable for discharge hospitalized for an additional ≥24 hours.

**Results:**

Of 274 patients included, 106 (38.7%) had pLOS (median age 65 years, 60.6% female, 69.0% black). Patients with pLOS had higher admission NIHSS than patients without pLOS (9 versus 5, *P* = 0.0010). A larger proportion of patients with pLOS developed an infection (*P* < 0.0001), and after adjusting for covariates, these patients had greater odds of poor short-term functional outcome (OR = 2.25, 95% CI 1.17–4.32, *P* = 0.0148). Adjusting for infection, the odds of patients with pLOS having poor short-term functional outcome were no longer significant (OR = 1.68, 95% CI 0.83–3.35, *P* = 0.1443).

**Conclusions:**

The contraction of a hospital-acquired infection was a significant predictor of pLOS and a contributor of poor short-term outcome following an ischemic stroke. Whether the cause or the consequence of pLOS, hospital-acquired infections are largely preventable and a target for reducing length of stay.

## 1. Introduction

Typical management of acute stroke has a variable duration, lasting from 5 to 14 days on average [1, 2]. Longer stays are more common in older patients [2–4] and those with more severe strokes [1], anterior circulation infarcts [2, 4], atrial fibrillation [1, 4], and hemorrhagic-type strokes [1, 2]. Longer stays also correlate with worse functional outcome and unfavorable discharge disposition in patients with acute ischemic stroke (AIS) [4, 5]. Koton et al. demonstrated a valid scoring tool to identify patients with stroke at risk for a hospitalization of ≥7 days at triage [1, 6]. Unfortunately, this tool was designed to predict prolonged hospitalization using common data elements collected at triage, failing to take into account complications that may occur during the inpatient stay.

Second, studies that have described in-hospital associations with pLOS defined length of stay by an absolute threshold, with some using a threshold of about 30 days [4, 7]. These studies do not compare differences between patients with and without pLOS. More importantly, they do not include patients with pLOS with a hospital course of <30 days—which represents the majority of stroke patients at U.S. centers [8].

In this study, we define pLOS without a threshold to better demonstrate its associations. We aim to identify in-hospital risk factors for pLOS and identify the driving force(s) for its association with poor functional outcome among patients with AIS [4]. Uncovering the underlying in-hospital risk factors and etiologies for pLOS may allow medical centers to develop better interventions to reduce pLOS.

## 2. Methods

We conducted a single-center retrospective analysis of patient information collected in a prospective stroke registry between July 2008 and December 2010 [9]. Patients were included unless they arrived >48 hours after time last seen normal, had an unknown last seen normal, experienced an in-hospital stroke, or did not have pLOS data documented. pLOS was defined as an extension in hospitalization for ≥24 hours more than would be necessary to stabilize the patient for discharge, as determined by the review of progress notes. Our definition depends on the patient’s neurologic stability for discharge rather than the completion of a stroke work-up for two reasons: (1) aspects of the stroke work-up may not be appropriate in acute management, and (2) the work-up itself may be a cause for pLOS. Determination of pLOS and the reasons for pLOS were documented prior to development of the aims of the present study using a case report form with three questions: (1) Was hospitalization prolonged due to complication? (2) Was hospitalization prolonged due to imaging or procedure? and (3) Was hospitalization prolonged due to securing disposition? Further chart audit by trained personnel, including a vascular neurologist, determined the specific cause of pLOS. We investigated admission, in-hospital, and outcome data elements associated with pLOS. Causes of pLOS were grouped into the following categories: neurologic complications, nonneurologic medical complications (including hospital-acquired infections [HAI]), delay due to imaging, delay due to procedure(s), and delay in disposition arrangement. HAI was distinguished from infection on admission via white blood cell count, clinical symptoms, and/or a positive culture for bacterium other than common contaminant. These categories were compared on admission, in-hospital, and functional outcome data elements.

We assessed the following outcome variables in our population: neurologic deterioration (defined as an increase in the National Institutes of Health Stroke Scale [NIHSS] by 2 or more points in a 24-hour period during hospital stay) [10], discharge NIHSS score, discharge modified Rankin Scale (mRS) score [11, 12], poor functional outcome at discharge (mRS >2), unfavorable discharge disposition (discharge to a facility other than inpatient rehabilitation or home), and in-hospital mortality.

Categorical data were compared using Pearson’s Chi-square test or Fisher’s exact test where appropriate. Continuous data (presented as mean/standard deviation for normally distributed data or median (range) for nonnormally distributed data) were compared using *t*-test for normally distributed data or Wilcoxon rank sum for nonnormally distributed data. Associations between exposures of interest and outcome measures were assessed using crude and adjusted logistic regression models. As this was an exploratory analysis, no adjustments were made for multiple comparisons [13]. All tests were performed at the α = 0.05 level.

## 3. Results

### 3.1. Demographics

Of the 274 patients included (median age 65 years, 60.6% female, 69.0% black), 106 (38.7%) had pLOS. The mean duration of hospitalization for patients without pLOS was 4 days compared to 9 days for patients with pLOS (*P* < 0.0001). Of baseline demographic measures, only baseline NIHSS differed significantly, with pLOS patients having a higher median score (9 versus 5, *P* = 0.0010). There were no significant differences in stroke etiology or infarct vascular distribution between patients with pLOS versus those without pLOS (Table 1).

### 3.2. In-Hospital Risk Factors

Thirty-six (34.9%) patients with pLOS had prolonged hospitalizations due to nonneurologic medical complications (e.g., acute kidney injury, acute respiratory distress syndrome, myocardial infarction). Fifteen (14.2%) patients with pLOS had extended hospitalizations due to HAI. Compared to all other patients in our population, patients with HAI were significantly older (median age 74 versus 64, *P* = 0.0036). Of patients with HAI, patients with pLOS were older (median age 81 versus 73, *P* = 0.0447) but had no other significant differences at baseline.

Among medical complications that prolong hospitalization, 3 of our patients with pLOS (2.8%) had extended stays due to pulmonary embolism, and 1 patient (0.9%) had an extended stay due to a deep vein thrombosis, despite routine venous thrombosis prophylaxis.

### 3.3. pLOS, Poor Outcomes, and Hospital-Acquired Infection

While patients with pLOS did not have higher proportions of in-hospital mortality (Table 2), patients with pLOS were at greater odds of neurologic deterioration compared to patients without pLOS (unadjusted OR = 3.172, 95% CI 1.88– 5.37, *P* < 0.0001). In terms of discharge measures, pLOS was significantly associated with poor short-term functional outcome (crude OR = 3.00, 95% CI 1.77–5.09, *P* < 0.0001), higher discharge NIHSS (median NIHSS 6 versus 2, *P* < 0.0001), and unfavorable discharge disposition (crude OR = 3.16, 95% CI 1.48–6.77, *P* = 0.0030). After adjusting for baseline NIHSS, age, and admission serum glucose, pLOS remained an independent predictor of poor short-term functional outcome (adjusted OR = 2.25, 95% CI 1.17–4.32, *P* = 0.0148) but not significantly associated with unfavorable discharge disposition (adjusted OR = 2.27, 95% CI 0.93–5.52, *P* = 0.0707). Once infection was added to the model, pLOS was no longer an independent predictor of short-term poor functional outcome (adjusted OR = 1.68, 95% CI 0.83–3.35, *P* = 0.1443) or unfavorable discharge disposition (adjusted OR = 1.60, 95% CI 0.60–4.25, *P* = 0.3444). Urinary tract infection (UTI) and pneumonia accounted for over 80% of HAI incidence.

Further, patients with the combination of pLOS and HAI were at greater odds of discharge NIHSS being greater than baseline NIHSS (crude OR = 5.58, 95% CI 2.34–13.28, *P* = 0.0001), discharge NIHSS >14 (crude OR = 8.27, 95% CI 3.21–21.27, *P* < 0.0001), poor short-term functional outcome (crude OR = 11.98, 95% CI 3.33–43.1, *P* = 0.0001), and unfavorable discharge disposition (crude OR = 6.04, 95% CI 1.99–18.28, *P* = 0.0015) when compared to patients with pLOS who did not develop a HAI.

Alternatively, among patients with HAI, pLOS was not significantly associated with short-term poor functional outcome (OR = 2.87, 95% CI 0.53–15.7, *P* = 0.2229) or unfavorable discharge disposition (OR=1.63, 95% CI 0.44–5.98, *P* = 0.4619) when compared to patients with HAI who did not have pLOS.

## 4. Discussion

In our study, nearly half of the patients with ischemic stroke were hospitalized for ≥24 hours more than necessary to stabilize them for discharge, with medical complications being the most contributory in-hospital cause. Patients with pLOS had significantly worse outcomes in nearly every measure. Of nonmodifiable risk factors, patients with pLOS had more severe neurologic dysfunction at baseline and were older than patients who did not experience pLOS, in keeping with previous reports [1, 3–5, 14]. Of modifiable risk factors, nonneurologic medical complications had the strongest association with pLOS. In particular, HAI significantly contributed to pLOS. In fact, the association between pLOS and short-term poor functional outcome (with adjustments for known predictors of poor outcome and mortality) was no longer statistically significant once adjusting for infection. Despite different approaches, our findings closely aligned with a previous study relating symptomatic UTIs in acute stroke to unfavorable discharge disposition [15]. Taken together, these results indicate that pLOS is not independently associated with poor functional outcome after stroke, but that HAI may be the driving force for poor functional outcome in these patients who have prolonged hospital stays. Further studies are needed to establish if there is a linear relationship with infections leading to pLOS and then leading to poor functional outcome or if it is pLOS leading to infection which then leads to poor functional outcome. In our study, UTI and pneumonia were the most frequent etiologies of HAI and accounted for the majority of infection incidences. These results support diligent monitoring of patients at risk for HAI; namely patients requiring intubation [16], catheterization [17], peripherial venous access [18], patients with more severe stroke [19], dysphagia [19, 20], and the elderly [20]. Taking appropriate measures to prevent infection (e.g., exchanging and/or removing unnecessary catheters [21]) could significantly curb pLOS and its complications.

Our study is not without limitations. The retrospective nature of our study limits the ability to determine causal relationships. Further, our small sample size may limit our ability to detect existing differences between groups. Among patients with pLOS due to infection (*n* = 14), we can safely argue that the infection prolonged hospitalization for these patients due to the methodology implemented in our data abstraction process. However, we cannot make the same statement for the rest of the patients with pLOS prior to infection. Infection in these patients may or may not have caused a secondary pLOS. Regardless, our data indicates that infection and pLOS are compounding variables that contribute to poor short-term outcome in AIS patients.

Despite its limitations, our study is unique in that we did not use an absolute threshold for pLOS (in days) as in prior studies on neurologic diseases [1, 4, 7, 22]. Our definition was tailored to the specific management of the patient, as some investigators have shown that an absolute threshold of pLOS following stroke may not be applicable to all patients at all centers [23].

In summary, our findings confirm the association between pLOS and poor short-term functional outcome [4, 14, 24]. Unlike other studies, which we attempted to parse the association between HAI and pLOS to indicate HAI, especially UTI and pneumonia, was a major modifiable risk factor driving poor functional outcome in patients with pLOS.

## Figures and Tables

**Table 1:** Admission demographic information.

	LOS not prolonged (*n* = 168)	LOS prolonged (*n* = 106)	*P* value
Age, median y; IQR	62; 54, 76	67; 57, 78	0.0952
Race, (%)			0.3994
Black	113 (67.3%)	76 (71.7%)	
White	51 (30.4%)	27 (25.5%)	
Other	2 (2.3%)	3 (2.8%)	
Gender, female (%)	97 (57.7%)	69 (65.1%)	0.2249
In-hospital stroke, (%)	1 (0.6%)	0 (0.0%)	0.6131
Delay from LSN to ED, median; IQR	237; 61–775	330; 77–728	0.4107
Past medical history, (%)			
Stroke	72 (42.9%)	47 (44.3%)	0.8095
Coronary artery disease	31 (18.6%)	28 (26.4%)	0.1245
Diabetes mellitus	55 (32.9%)	39 (37.5%)	0.4425
Hypertension	131 (78.4%)	87 (83.6%)	0.2929
Atrial fibrillation	17 (10.2%)	8 (7.7%)	0.4820
Dyslipidemia	76 (45.8%)	42 (40.4%)	0.3841
Admission NIHSS, median; IQR	5; 2, 13.5	9; 4, 17	0.0010
Admission glucose, median mg/dL; IQR	114; 95, 148	121; 100, 148	0.2248
Admission hematocrit, median%; IQR	39.3; 35.9, 42.5	39.4; 35.3, 43.3	0.9477
TOAST, (%)			0.1078
Cardioembolic	45 (26.8%)	30 (28.3%)	
Large vessel	35 (20.8%)	35 (33.0%)	
Small vessel	37 (22.0%)	21 (19.8%)	
Unknown etiology	35 (20.8%)	17 (16.0%)	
More than one etiology	7 (4.2%)	1 (0.9%)	
Stroke Location, (%)			
MCA territory	109 (64.9%)	64 (60.4%)	0.4517
ACA territory	31 (18.5%)	11 (10.4%)	0.0708
PCA territory	30 (17.9%)	13 (12.3%)	0.2151
Other territory	27 (16.1%)	12 (11.3%)	0.2730
Treatment with IV tPA, (%)	64 (38.1%)	35 (33.0%)	0.3943

LOS: length of stay; IQR: interquartile range; LSN: last seen normal; ED: emergency department; NIHSS: National Institutes of Health Stroke Scale score; TOAST: Trial of org 10172 in acute stroke treatment; MCA: middle cerebral artery; ACA: anterior cerebral artery; PCA: posterior cerebral artery; IV tPA: intravenous tissue plasminogen activator.

**Table 2:** Outcome measures according to whether or not LOS was prolonged.

	LOS not prolonged (*n* = 168)	LOS prolonged (*n* = 106)	*P* value
In-hospital infection, (%)	20 (11.9%)	49 (46.2%)	<0.0001
Pneumonia	6 (3.6%)	19 (18.1%)	<0.0001
UTI	11 (6.7%)	34 (32.1%)	<0.0001
Other	5 (3.1%)	10 (9.4%)	0.0245
Neurologic deterioration, (%)	38 (22.6%)	51 (48.1%)	<0.0001
Discharge NIHSS, median; IQR	2; 0, 7	6; 2, 16	<0.0001
Discharge mRS, median; IQR	2; 1, 4	4; 2, 5	<0.0001
Poor functional outcome*, (%)	79 (48.2%)	78 (73.6%)	<0.0001
Unfavorable discharge disposition**, (%)	12 (7.8%)	21 (21.2%)	0.0021
Discharge disposition			<0.0001
Discharge to home	101 (66.0%)	30 (30.3%)	
Discharge to inpatient rehabilitation	40 (26.1%)	48 (48.5%)	
Discharge to skilled nursing facility	4 (2.6%)	11 (11.1%)	
Discharge to long-term acute care facility	1 (0.6%)	5 (5.1%)	
Discharge to hospice	5 (3.3%)	5 (5.1%)	
Length of stay, median days (IQR)	4; 2, 6	9; 4, 15	<0.0001
In-hospital mortality, (%)	14 (8.3%)	7 (6.6%)	0.6002

LOS: length of stay; NIHSS: National Institutes of Health Stroke Scale score; IQR: interquartile range; mRS: modified Rankin Scale score.

* Poor functional outcome is defined as discharge mRS >2.

** Unfavorable discharge disposition is defined as discharge to a facility other than home or inpatient rehabilitation.
